# Evaluation of vitamin D biosynthesis and pathway target genes reveals *UGT2A1/2* and *EGFR* polymorphisms associated with epithelial ovarian cancer in African American Women

**DOI:** 10.1002/cam4.1996

**Published:** 2019-04-18

**Authors:** Delores J. Grant, Ani Manichaikul, Anthony J. Alberg, Elisa V. Bandera, Jill Barnholtz‐Sloan, Melissa Bondy, Michele L. Cote, Ellen Funkhouser, Patricia G. Moorman, Lauren C. Peres, Edward S. Peters, Ann G. Schwartz, Paul D. Terry, Xin‐Qun Wang, Temitope O. Keku, Cathrine Hoyo, Andrew Berchuck, Dale P. Sandler, Jack A. Taylor, Katie M. O’Brien, Digna R. Velez Edwards, Todd L. Edwards, Alicia Beeghly‐Fadiel, Nicolas Wentzensen, Celeste Leigh Pearce, Anna H. Wu, Alice S. Whittemore, Valerie McGuire, Weiva Sieh, Joseph H. Rothstein, Francesmary Modugno, Roberta Ness, Kirsten Moysich, Mary Anne Rossing, Jennifer A. Doherty, Thomas A. Sellers, Jennifer B. Permuth‐Way, Alvaro N. Monteiro, Douglas A. Levine, Veronica Wendy Setiawan, Christopher A. Haiman, Loic LeMarchand, Lynne R. Wilkens, Beth Y. Karlan, Usha Menon, Susan Ramus, Simon Gayther, Aleksandra Gentry‐Maharaj, Kathryn L. Terry, Daniel W. Cramer, Ellen L. Goode, Melissa C. Larson, Scott H. Kaufmann, Rikki Cannioto, Kunle Odunsi, John L. Etter, Ruea‐Yea Huang, Marcus Q. Bernardini, Alicia A. Tone, Taymaa May, Marc T. Goodman, Pamela J. Thompson, Michael E. Carney, Shelley S. Tworoger, Elizabeth M. Poole, Diether Lambrechts, Ignace Vergote, Adriaan Vanderstichele, Els Van Nieuwenhuysen, Hoda Anton‐Culver, Argyrios Ziogas, James D. Brenton, Line Bjorge, Helga B. Salvensen, Lambertus A. Kiemeney, Leon F. A. G. Massuger, Tanja Pejovic, Amanda Bruegl, Melissa Moffitt, Linda Cook, Nhu D. Le, Angela Brooks‐Wilson, Linda E. Kelemen, Paul D.P. Pharoah, Honglin Song, Ian Campbell, Diana Eccles, Anna DeFazio, Catherine J. Kennedy, Joellen M. Schildkraut

**Affiliations:** ^1^ Department of Biological and Biomedical Sciences, Cancer Research Program JLC‐Biomedical/Biotechnology Research Institute, North Carolina Central University Durham North Carolina; ^2^ Center for Public Health Genomics University of Virginia Charlottesville Virginia; ^3^ Department of Epidemiology and Biostatistics, Arnold School of Public Health University of South Carolina Columbia South Carolina; ^4^ Department of Population Science Rutgers Cancer Institute of New Jersey New Brunswick New Jersey; ^5^ Case Comprehensive Cancer Center Case Western Reserve University School of Medicine Cleveland Ohio; ^6^ Cancer Prevention and Population Sciences Program Baylor College of Medicine Houston Texas; ^7^ Department of Oncology and the Karmanos Cancer Institute Population Studies and Disparities Research Program Wayne State University School of Medicine Detroit Michigan; ^8^ Division of Preventive Medicine University of Alabama at Birmingham Birmingham Alabama; ^9^ Department of Community and Family Medicine Duke University Medical Center Durham North Carolina; ^10^ Epidemiology Program Louisiana State University Health Sciences Center School of Public Health New Orleans Louisisana; ^11^ Department of Medicine University of Tennessee Medical Center – Knoxville Knoxville Tennessee; ^12^ Department of Public Health Sciences University of Virginia Charlottesville Virginia; ^13^ Departments of Medicine and Nutrition, Division of Gastroenterology and Hepatology University of North Carolina at Chapel Hill Chapel Hill North Carolina; ^14^ Department of Biological Sciences North Carolina State University Raleigh North Carolina; ^15^ Department of Obstetrics and Gynecology Duke University Medical Center Durham North Carolina; ^16^ Epidemiology Branch, Division of Intramural Research National Institute of Environmental Health Sciences, National Institutes of Health Research Triangle Park North Carolina; ^17^ Vanderbilt Epidemiology Center, Center for Human Genetics Research, Department of Obstetrics and Gynecology Vanderbilt University Medical Center Nashville Tennessee; ^18^ Division of Epidemiology, Center for Human Genetics Research, Department of Medicine Vanderbilt University Medical Center Nashville Tennessee; ^19^ Division of Epidemiology, Department of Medicine, Vanderbilt Epidemiology Center Institute for Medicine and Public Health, Vanderbilt University Medical Center Nashville Tennessee; ^20^ Division of Cancer Epidemiology and Genetics National Cancer Institute Bethesda Maryland; ^21^ Department of Epidemiology University of Michigan School of Public Health Ann Arbor Michigan; ^22^ Department of Preventive Medicine, Keck School of Medicine University of Southern California Norris Comprehensive Cancer Center Los Angeles California; ^23^ Department of Health Research and Policy Stanford University School of Medicine Stanford California; ^24^ Department of Biomedical Data Science Stanford University School of Medicine Stanford California; ^25^ Department of Population Health Science and Policy Icahn School of Medicine at Mount Sinai New York New York; ^26^ Department of Genetics and Genomic Sciences Icahn School of Medicine at Mount Sinai New York New York; ^27^ Division of Gynecologic Oncology, Department of Obstetrics, Gynecology and Reproductive Sciences University of Pittsburgh School of Medicine Pittsburgh Pennsylvania; ^28^ Department of Epidemiology University of Pittsburgh Graduate School of Public Health Pittsburgh Pennsylvania; ^29^ Ovarian Cancer Center of Excellence, Womens Cancer Research Program Magee‐Womens Research Institute and University of Pittsburgh Cancer Institute Pittsburgh Pennsylvania; ^30^ The University of Texas School of Public Health Houston Texas; ^31^ Department of Cancer Prevention and Control Roswell Park Cancer Institute Buffalo New York; ^32^ Program in Epidemiology, Division of Public Health Sciences Fred Hutchinson Cancer Research Center Seattle Washington; ^33^ Department of Epidemiology University of Washington Seattle Washington; ^34^ Department of Population Health Sciences Huntsman Cancer Institute, University of Utah Salt Lake City, Utah; ^35^ Department of Cancer Epidemiology Moffitt Cancer Center Tampa Florida; ^36^ Gynecology Service, Department of Surgery Memorial Sloan Kettering Cancer Center New York New York; ^37^ Gynecologic Oncology, Laura and Isaac Pearlmutter Cancer Center New York University Langone Medical Center New York New York; ^38^ University of Southern California Norris Comprehensive Cancer Center Los Angeles California; ^39^ University of Hawaii Cancer Center Honolulu Hawaii; ^40^ Cancer Epidemiology Program University of Hawaii Cancer Center Hawaii; ^41^ Women's Cancer Program Samuel Oschin Comprehensive Cancer Institute, Cedars‐Sinai Medical Center Los Angeles California; ^42^ MRC CTU at UCL, Institute of Clinical Trials and Methodology University College London London UK; ^43^ School of Women's and Children's Health University of New South Wales New South Wales Australia; ^44^ The Kinghorn Cancer Centre Garvan Institute of Medical Research Darlinghurst New South Wales Australia; ^45^ Center for Cancer Prevention and Translational Genomics Samuel Oschin Comprehensive Cancer Institute, Cedars‐Sinai Medical Center Los Angeles California; ^46^ Department of Biomedical Sciences Cedars‐Sinai Medical Center Los Angeles California; ^47^ Obstetrics and Gynecology Epidemiology Center Brigham and Women's Hospital Boston Massachusetts; ^48^ Harvard T. H. Chan School of Public Health Boston Massauchusetts; ^49^ Department of Health Science Research, Division of Epidemiology Mayo Clinic Rochester Minnesota; ^50^ Department of Health Science Research, Division of Biomedical Statistics and Informatics Mayo Clinic Rochester Minnesota; ^51^ Departments of Medicine and Pharmacology Mayo Clinic Rochester Minnesota; ^52^ Cancer Pathology & Prevention, Division of Cancer Prevention and Population Sciences Roswell Park Cancer Institute Buffalo New York; ^53^ Department of Gynecological Oncology Roswell Park Cancer Institute Buffalo New York; ^54^ Center For Immunotherapy Roswell Park Cancer Institute Buffalo New York; ^55^ Division of Gynecologic Oncology Princess Margaret Hospital, University Health Network Toronto Ontario Canada; ^56^ Cancer Prevention and Control Samuel Oschin Comprehensive Cancer Institute, Cedars‐Sinai Medical Center Los Angeles California; ^57^ Department of Biomedical Sciences Community and Population Health Research Institute, Cedars‐Sinai Medical Center Los Angeles California; ^58^ Department of Obstetrics and Gynecology John A. Burns School of Medicine, University of Hawaii Honolulu Hawaii; ^59^ Channing Division of Network Medicine Brigham and Women's Hospital and Harvard Medical School Boston Massachusetts; ^60^ Biostatistics, Sanofi Genzyme Boston Massachusetts; ^61^ Vesalius Research Center, VIB Leuven Belgium; ^62^ Laboratory for Translational Genetics, Department of Oncology University of Leuven Belgium; ^63^ Division of Gynecologic Oncology, Department of Obstetrics and Gynaecology and Leuven Cancer Institute University Hospitals Leuven Leuven Belgium; ^64^ Department of Epidemiology, Director of Genetic Epidemiology Research Institute, Center for Cancer Genetics Research & Prevention, School of Medicine University of California Irvine Irvine California; ^65^ Department of Epidemiology University of California Irvine Irvine California; ^66^ Cancer Research UK Cambridge Institute University of Cambridge Cambridge UK; ^67^ Department of Gynecology and Obstetrics Haukeland University Hospital Bergen Norway; ^68^ Centre for Cancer Biomarkers, Department of Clinical Science University of Bergen Bergen Norway; ^69^ Radboud University Medical Center Radboud Institute for Health Sciences Nijmegen Netherlands; ^70^ Department of Gynaecology, Radboud University Medical Center Radboud Institute for Molecular Life sciences Nijmegen The Netherlands; ^71^ Department of Obstetrics & Gynecology Oregon Health & Science University Portland Oregon; ^72^ Knight Cancer Institute, Oregon Health & Science University Portland Oregon; ^73^ Division of Epidemiology and Biostatistics, Department of Internal Medicine University of New Mexico Albuquerque New Mexico; ^74^ Cancer Control Research, British Columbia Cancer Agency Vancouver British Columbia Canada; ^75^ Canada's Michael Smith Genome Sciences Centre British Columbia Cancer Agency Vancouver British Columbia Canada; ^76^ Department of Biomedical Physiology and Kinesiology Simon Fraser University Burnaby British Columbia Canada; ^77^ Hollings Cancer Center and Department of Public Health Sciences Medical University of South Carolina Charleston South Carolina; ^78^ Strangeways Research laboratory, Department of Oncology, Department of Public Health and Primary Care University of Cambridge Cambridge UK; ^79^ Strangeways Research Laboratory, Department of Oncology University of Cambridge Cambridge UK; ^80^ Cancer Genetics Laboratory, Research Division Peter MacCallum Cancer Centre Victoria Australia; ^81^ Department of Pathology University of Melbourne Parkville Victoria Australia; ^82^ Faculty of Medicine University of Southampton Southampton UK; ^83^ Centre for Cancer Research The Westmead Institute for Medical Research, The University of Sydney Sydney New South Wales Australia; ^84^ Department of Gynaecological Oncology Westmead Hospital Sydney New South Wales Australia

**Keywords:** African ancestry risk, genetic association, ovarian cancer, vitamin D pathway

## Abstract

An association between genetic variants in the vitamin D receptor (*VDR*) gene and epithelial ovarian cancer (EOC) was previously reported in women of African ancestry (AA). We sought to examine associations between genetic variants in VDR and additional genes from vitamin D biosynthesis and pathway targets (*EGFR, UGT1A, UGT2A1/2, UGT2B, CYP3A4/5*, *CYP2R1*, *CYP27B1*, *CYP24A1*, *CYP11A1*, and *GC*). Genotyping was performed using the custom‐designed 533,631 SNP Illumina OncoArray with imputation to the 1,000 Genomes Phase 3 v5 reference set in 755 EOC cases, including 537 high‐grade serous (HGSOC), and 1,235 controls. All subjects are of African ancestry (AA). Logistic regression was performed to estimate odds ratios (OR) and 95% confidence intervals (CI). We further evaluated statistical significance of selected SNPs using the Bayesian False Discovery Probability (BFDP). A significant association with EOC was identified in the *UGT2A1/2* region for the SNP rs10017134 (per allele OR = 1.4, 95% CI = 1.2‐1.7, *P* = 1.2 × 10^−6^, BFDP = 0.02); and an association with HGSOC was identified in the *EGFR* region for the SNP rs114972508 (per allele OR = 2.3, 95% CI = 1.6‐3.4, *P* = 1.6 × 10^−5^, BFDP = 0.29) and in the *UGT2A1/2* region again for rs1017134 (per allele OR = 1.4, 95% CI = 1.2‐1.7, *P* = 2.3 × 10^−5^, BFDP = 0.23). Genetic variants in the *EGFR* and *UGT2A1/2* may increase susceptibility of EOC in AA women. Future studies to validate these findings are warranted. Alterations in *EGFR* and *UGT2A1/2* could perturb enzyme efficacy, proliferation in ovaries, impact and mark susceptibility to EOC.

## 1 INTRODUCTION

Women of African ancestry (AA) have the lowest incidence of ovarian cancer worldwide, but they tend to present with more advanced tumors and have lower 5‐year survival (35%) compared to women of European descent (47%) in nearly every cancer subtype.^1^, ^2^ Compared to Caucasian women, there have been fewer published studies investigating the association between common risk factors, such as tubal ligation, use of hormonal contraceptives, obesity, body powder and dietary patterns, and ovarian cancer risk in AA.^1^, ^3^, ^4^, ^5^, ^6^, ^7^, ^8^, ^9^ Moreover, the investigation of genetic susceptibility to epithelial ovarian cancer (EOC) in AA has not been comprehensive. The limited assessment of genetic susceptibility among AA is in modest sized study populations of candidate genes including the repeat polymorphisms of the androgen receptor (*AR*), vitamin D receptor (*VDR*) and cellular transport genes, where an association with risk of ovarian cancer was observed.^10^, ^11^, ^12^

The vitamin D receptor mediates the regulation of a pleotropic cascade of physiological responses; including those involved in phase I and phase II detoxification and the epidermal growth factor receptor (*EGFR*) proliferation pathways in ovarian and other cancer cell lines; through VDR/DNA interactions and bioavailability of vitamin D.^13^, ^14^, ^15^, ^16^, ^17^ A *VDR* variant, rs7305032, was associated with ovarian cancer in 125 cases and 155 controls of AA but other observations were limited because of small sample size.^11^ Moreover, known genetic variations in the VDR/vitamin D biosynthesis and pathway target genes have been implicated in AA disease risk. Therefore an objective of this study was to assess those variants in ovarian cancer in women of African ancestry in a large sample.

Using a candidate gene approach, SNPs were selected from genes involved in vitamin D biosynthesis and metabolism; and putative targets of VDR regulation. Genes of the vitamin D biosynthesis pathways included cytochrome P450s:*CYP2R1*, *CYP27B1*, *CYP24A1*, *CYP11A1*, and group‐specific component‐vitamin D‐binding protein (GC) which collectively are responsible for the homeostatic control and bioavailability of vitamin D.^18^, ^19^, ^20^, ^21^, ^22^, ^23^ The candidate genes involved in vitamin D metabolic processes included *CYP3A4/5* and UDP‐glucuronosyltransferase 1A (*UGT1A*) locus members. They are responsible for glucuronidation and hydroxylation of the biologically active and circulatory forms of vitamin D. These genes are also inclusive of candidates regulated by vitamin D/VDR binding and included *CYP3A4/5*, *UGT1A* locus members, *EGFR* and UDP‐glucuronosyltransferase 2 (*UGT2*) locus members; that are associated, in part, with other cancers in AA individuals.^24^, ^25^, ^26^, ^27^, ^28^, ^29^, ^30^, ^31^, ^32^, ^33^, ^34^, ^35^, ^36^, ^37^, ^38^, ^39^ Thus, variants in *VDR* and additional genes from vitamin D biosynthesis and pathway targets are viable candidates to investigate the genetic underpinnings of ovarian cancer risk in women of African descent.

In this study, SNPs from 11 gene regions: *VDR*, *EGFR, UGT1A, UGT2A1/2, UGT2B, CYP3A4/5*, *CYP2R1*, *CYP27B1*, *CYP24A1*, *CYP11A1*, and *GC*, were genotyped, imputed then assessed for risk of EOC and high grade serous ovarian cancer (HGSOC) in cases and controls of AA from the African American Cancer Epidemiology Study (AACES)^40^ and the Ovarian Cancer Association Consortium (OCAC).^41^

## 2 MATERIALS AND METHODS

### 2.1 Study populations

The Genetic Associations and Mechanisms in Oncology (GAME‐ON) project comprised 63, mostly, case‐control studies from four continents (North America, Europe, Asia and Australia). Only 32 studies contributed subjects of African Ancestry, including AACES and studies in OCAC, and were included in the current analysis (Supplemental Table S1). AACES, previously described elsewhere,^40^ is a multi‐center population‐based case‐control study of newly diagnosed invasive EOC in African American women that enrolled study subjects between 2010 and 2015. Established in 2005, OCAC is an international consortium focused on genetic association and pooled risk factor analyses. The current analyses included 1990 samples: 1235 controls and 755 invasive EOC cases who passed quality control filters, all of whom were AA. The majority of the EOC cases were HGSOC (n = 537, 71%), followed by 49 mucinous cases (7%), 28 endometrioid cases (4%), 23 clear cell cases (3%), 12 mixed histology (2%) and 53 other (7%). All subjects included in this analysis provided written informed consent as well as data and blood samples under ethically approved protocols.

### 2.2 Genotyping, ancestry analysis and quality control

Genotyping of AA women from OCAC was completed using the custom‐designed 533,631 SNP array, the Illumina OncoArray. Sample level quality control included restriction to females, filter on call rate >95%, heterozygosity (either too big or too small), removal of ineligible samples, and relationship inference to check for unexpected first degree relatives. SNP level quality control included filter on call rate >95%, and Hardy–Weinberg Equilibrium *p*‐value >1 × 10^−5^. After applying these procedures, 471,780 SNPs remained.

Intercontinental ancestry was calculated for the OCAC and AACES samples using the software package FastPop^42^ that was developed specifically for the OncoArray Consortium. Only the African ancestry samples defined as having >50% AA were used for the present analyses reported here. Seventy‐seven cases and 120 controls were omitted due to African ancestry <50% and one gender mismatch. Principal components computed using FastPop were further used to adjust for population structure in our analyses.

### 2.3 Genotype imputation analysis

Using the genotyped SNPs that passed quality control, haplotypes were phased using SHAPEIT v2 followed by imputation to the 1,000 Genomes Phase 3 v5 reference set using Minimac3.

### 2.4 Gene region and SNP selection

Eleven gene regions were defined based on human genome build 37. SNPs within the selected regions were filtered on imputation quality score (minimac imputation R‐squared) >0.5 for imputed SNPs, or Hardy–Weinberg Equilibrium *p*‐value >1.0 × 10^−5^ for genotyped SNPs. Quantile‐quantile plots on the EOC and HGSOC dataset (Manichaikul et al, unpublished) have lambdas of 1.01 each within normal range.^43^ The imputation quality scores for significant SNPs are provided. We further applied filters on effective heterozygosity count (HC) > 30. After applying filters, the following number of SNPs remaining in each of the selected gene regions for EOC was: 288 in *VDR*, 433 in *UGT2A1/2*, 6302 in *UGT2B*, 919 in *UGT1A*, 963 in *EGFR*, 17 in *CYP2R1*, 4 in *CYP27B1*, 113 in *CYP24A1*, 90 in *CYP11A1*, 411 in *CYP3A4/5* and 296 in *GC*. For selected regions for HGSOC analysis, the number of SNPs was: 234 in *VDR*, 413 in *UGT2A1/2*, 5674 in *UGT2B*, 833 in *UGT1A*, 824 in *EGFR*, 15 in *CYP2R1*, 4 in *CYP27B1*, 106 in *CYP24A1*, 82 in *CYP11A1*, 375 in *CYP3A4/5* and 282 in *GC.*

### 2.5 Statistical analysis

Genetic association testing was carried out with adjustment for two principal components (PCs) of ancestry using a logistic regression model that accounts for genotype uncertainty under a score test as implemented in SNPTEST v2.5.2 to estimate odds ratios (OR) and 95% confidence intervals (CI). For each gene region, we applied a gene‐specific Bonferroni‐threshold for statistical significance defined as 0.05/number of SNPs examined for that gene. We further assessed the main results with an alternative to the Bonferroni threshold using the Bayesian False Discovery Probability (BFDP) which provides the posterior probability of a false discovery based on a given prior probability of nonnull association at a given SNP.^44^ For this study we specified a prior probability of association at each SNP under investigation based on the total number of SNPs within each candidate gene region as 0.5 × 1/(N_SNP_/3) where N_SNP_ represents the number of SNPs in the given candidate gene region. We considered N_SNP_/3 to be an approximation of the effective number of independent SNPs within in each gene region, taking into account the fact that many SNPs will be correlated due to linkage disequilibrium. Accordingly, the specified prior indicates a 50% chance of true discovery within each gene region, with the prior probability of nonnull association distributed randomly among all SNPs within the region. In order to avoid spurious positive associations, we applied a filter on effective (HC) > 30 in each of cases and controls. Here, HC is defined as N × MAF ×  (1‐MAF) for each SNP, N represents the sample size (either the number of cases or the number of controls), and MAF represents the SNP minor allele frequency. Based on 755 EOC cases and 537 HGSOC cases, respectively, applying this filter equates to applying a SNP MAF filter of 4.2% and 6% in analysis of EOC and HGSOC, respectively. Statistical power calculations for AA study participants and Caucasians are included in Supplemental Tables S2 and S3.

## 3 RESULTS

### 3.1 VDR pathway gene regions and risk of EOC

SNPs from 11 gene regions (CYP3A4/5,*CYP2R1*, *CYP27B1*, *CYP24A1*, *CYP11A1*, *EGFR*, *GC*, *UGT1A*, *UGT2A1/2*, *UGT2B and VDR*) from VDR biosynthesis and pathway targets were assessed for association with EOC (Supplemental Table S4). The top associations are reported in Table 1. Individuals carrying the major allele of SNP rs10017134 of the *UGT2A1/2* gene region had an increased odds of EOC when corrected for multiple comparisons (OR = 1.4, 95% CI = 1.2‐1.7, *P* = 1.2 × 10^−6^). The BFDP for rs10017134 of 0.020 corresponds to 98% posterior probability of nonnull association for this SNP. Significant associations with EOC were also observed for *UGT2A1/2* SNPs, rs2288741 and rs11939884. The variants are found in both *UGT2A1* and *UGT2A2* as the genes share common exons 2 through 6.^45^ Supplemental Table S5 summarizes other notable (*P* < 0.01) SNP associations with EOC in the OncoArray analysis.

**Table 1:** Top SNP *P*‐values from gene regions associated with EOC in African American OncoArray analysis

SNP ID (Effect/other allele) *Nearest gene(s)*	Effect Allele Frequency	N	OR	95% CI	*P*‐value	Bayesian False Discovery Probability (BFDP)	Imputation quality
rs10017134 (C/T) UGT2A1/2^a^, ^b^	0.73	1990	1.4	1.2‐1.7	1.2 × 10^−6^	0.020	0.998
rs2288741 (T/G) UGT2A1/2^b^	0.73	1990	1.4	1.2‐1.6	1.9 × 10^−6^	—	—
rs11939884 (T/G) UGT2A1/2^a^, ^b^	0.14	1990	0.7	0.5‐0.8	1.7 × 10^−6^	—	—

a Imputed.

b Bonferroni correction was applied to adjust for multiple SNPs comparisons. There were 433 SNPs in UGT2A1/2 gene. BFDP is reported based on a prior probability of association (pi0) equal to 0.6 * 1/(Number of SNPs/3).

### 3.2 VDR pathway gene regions and risk of HGSOC

SNPs from the 10 gene regions from VDR biosynthesis and pathway targets were assessed for association with HGSOC (Supplemental Table S4). The top associations are in reported in Table 2. Individuals carrying the minor allele of *EGFR* SNP rs114972508 had more than twofold increased odds of HGSOC (OR = 2.3, 95% CI = 1.2‐3.4, *P* = 1.6 × 10^−5^) (Table 2). The posterior BFDP is 29% for SNP rs114972508 corresponds to 71% posterior probability of nonnull association. SNP rs10017134 of the *UGT2A1/2* gene region also showed association with HGSOC (OR = 1.4, 95% CI = 1.2‐1.7, *P* = 2.3 × 10^−5^) (Table 2). The posterior BFDP is 22.8%. Supplemental Table S6 summarizes other notable (*P* < 0.01) SNP associations with HGSOC in the OncoArray analysis.

**Table 2:** Top SNP *P*‐values from gene regions associated with HGSOC in African American OncoArray analysis

SNP ID (Effect/other allele) *Nearest gene(s)*	Effect Allele Frequency	N	OR	95% CI	*P*‐value	Bayesian False Discovery Probability (BFDP)	Imputation quality
rs114972508 (T/C) EGFR^a^, ^b^	0.04	1772	2.3	1.2‐3.4	1.6 × 10^−5^	0.293	0.890
rs10017134 (C/T) UGT2A1/2^a^,^b^	0.72	1772	1.4	1.2‐1.7	2.3 × 10^−5^	0.228	0.998
rs2288741 (T/G) UGT2A1/2^b^	0.72	1772	1.4	1.2‐1.7	3.1 × 10^−5^	—	—

a Imputed.

b Bonferroni correction was applied to adjust for multiple SNPs comparisons. There were 824 SNPs in EGFR gene, and 413 SNPs in UGT2A1/2 gene. BFDP is reported based on a prior probability of association (pi0) equal to 0.5 * 1/(Number of SNPs/3).

## 4 DISCUSSION

Few studies have investigated the genetic susceptibility for ovarian cancer among women of African descent. The assessment of candidate SNPs from chromosomal regions that contain genes regulated by VDR activity provides some evidence of association with EOC risk. The notable findings from this analysis show, for the first time, that risk assessments of variants in the *UGT2A1/2* and *EGFR* gene regions are suggestive of associations with EOC and HGSOC. The results also demonstrate evidence of associations for other SNPs from the candidate gene regions with EOC and HGSOC. Although the candidate SNPs are located in intronic regions there is ample evidence that many gene regulatory regions are present in those regions including encoded microRNAs, alternate splice sites, and cis‐regulatory modules and transcription factors binding sites.^46^, ^47^, ^48^ In addition, recent studies have shown using targeted RNAseq analysis that there are numerous splice variants of the *UGT* genes.^49^

The *UGT2A1* and *2A2* genes are distinguished by unique first exons joined to common exons 2‐6 and are located downstream of *UGT2B4* on chromosome 4.^45^ *UGT2A* transcripts have been detected in several extrahepatic tissues such as the lung, trachea, larynx, intestine, pancreas, and kidney.^50^ UGT2A1 is an extrahepatic enzyme that is expressed mainly in the nasal epithelium, catalyzing the glucuronidation of testosterone and epitestosterone at considerable rates and has similar kinetics as the *UGT2B* gene family members.^51^ There are reports that this enzyme also has activity toward estrogen metabolites epiestradiol and β‐estradiol.^52^ UGT2A1 has exhibited highest expression in the lung, followed by trachea, tonsil, larynx, colon, olfactory.^53^ *UGT2A2* mRNA expression was reported in fetal and adult nasal mucosa tissues.^54^ However, unlike UGT2A1, other expression analyses suggested that wild‐type UGT2A2 had the highest expression in the breast, followed by trachea, larynx, and kidney.^55^

Neither the *UGT2A1* gene, nor UGT2A2 expression have been examined in ovarian tissue. However, VDR ChIPseq peak locations have been identified 430 kb downstream of the *UGT2A1/2* locus in experiments with THP‐1 cells treated with 1a,25(OH)_2_D_3_, the biologically active form of the vitamin D hormone, suggestive of a regulatory role for vitamin D.^56^ Splice variants found in *UGT2A1/2* that are highly conserved among both *UGT1A* and *UGT2* gene families have been implicated in altered glucuronidation activity against tobacco carcinogenesis.^49^, ^53^, ^55^, ^57^ Two of the *UGT2A1* SNPs associated with EOC and HGSOC in this study are intron variants (rs10017134 and rs2288741) while the third (rs11939884) is a 3’ UTR variant. It is probable that these variants alter enzyme function in target tissues including ovarian and/or alter risk in AA smokers. Of note, cigarette smoking has been found to be associated with the risk of mucinous EOC, but not HGSOC among Caucasian women.^58^ Moreover, providing some plausibility for the mechanism of the observed SNP association, a recent report suggests that cigarette smoking may be associated with serous EOC among African American women although a dose‐response relationship was not observed.^59^ The association of genes from the *UGT* superfamily with ovarian cancer in AA is consistent with significant associations observed for Caucasian women for *UGT1A*.^12^ However in this study, no association was observed for AA samples with SNPs with a MAF of 0.42 for the risk allele while associations were observed in Caucasians with SNPs with a MAF of 0.07. Some but not all MAFs for the relationships observed in this study differ by race so it is unlikely to explain racial differences in risk.

The *EGFR* gene product has been a chemotherapeutic target for EOC since overexpression has been linked to poor prognosis in ovarian cancer patients.^60^, ^61^, ^62^ The signaling pathway for EGFR is mediated by ligands including the epidermal growth factor in the regulation of cell proliferation, differentiation and apoptosis in normal cells. Research into the mechanisms of EGFR overexpression has focused on mutations and amplifications in the coding region of the gene containing the receptor tyrosine kinase domain.^63^ However, few studies on SNP variants in this region have been linked to EOC or other ovarian cancer histologic subtypes.^61^, ^63^ *EGFR* SNP rs114972508 is located in intron 1 of the *EGFR* gene. The location of the SNP is approximately 70 kb upstream of a *VDR* binding site also within *EGFR* intron 1 that has been shown experimentally to down regulate *EGFR* expression and proliferative function.^15^ Perhaps changes in the intron sequences may impact *EGFR* function and subsequently be as critical to cellular homeostasis as the receptor tyrosine function that has been extensively researched. Thus, *EGFR* SNPs could be abrogating vitamin D hormone regulation of ovarian cell proliferation and increasing susceptibility for the development of HGSOC in AA women.

Although we were unable to confirm the association between previously identified *VDR* variants and risk of EOC, a recent case‐control study of women of European ancestry (10,065 cases, 21,654 controls) showed that SNPs associated with decreased circulating 25‐hydroxyvitamin D were associated with ovarian cancer and HGSOC^64^ while another study showed that AA women exposed to increased sunlight had a decreased risk for ovarian cancer.^8^ These observations suggest that other mechanisms affecting vitamin D hormone activity independent of the VDR may be important in ovarian cancer etiology.

The main observations in the current study result from associations of imputations of genotyped SNPs but independent of *VDR* variant association with EOC and HGSOC. The *VDR* SNPs previously observed to be associated with the risk of EOC,^11^ including rs7975232 and rs7305032, were not associated with risk of EOC in the current study (Supplemental Table S7). A look up of the significant study SNPs in archived OCAC data on Caucacians shows no significant associations for the *UGT2A1/2* SNPs. Data on the *EGFR* SNP were not available (Supplemental Table S8). Other *VDR* SNPs showed nominal (nonBonferroni corrected) associations with EOC but not with HGSOC (Supplemental Table S7). Although the largest study to date of genetic association with EOC in AA, the modest sample size remains a limitation of the current study and therefore some of the nominal SNP associations may be a result of inadequate power. The analyses are underpowered for discovery analysis across the selected gene regions and important associations may have been missed, nonetheless, we still found significantly associations with EOC and HGSOC. Several suggestive and nominal SNP associations (outside of Bonferroni significance) may provide some insight and consideration for future experimental studies to further explore the relevance of vitamin D biosynthesis and pathway target genes. Larger studies of AA are warranted to clarify these finding.

In summary, this study reports, for the first time, an association between *EGFR* and *UGT2A1/2* variants with ovarian cancer risk in AA women. These gene variants could perturb cell proliferation and enzyme efficacy in ovaries and impact susceptibility to ovarian cancer by altering growth and intercellular hormone metabolism. Future studies are needed to validate the associations of the imputed SNPs and to determine their impact on cancer development. Currently, there are no published reports of population studies of *UGT2A1/2* polymorphisms in Europeans or other racially distinct groups in larger sample sizes than this AA study that would allow intricate gene‐environment analysis. At this present time, there is only limited evidence that *UGT2B* gene region variants may be associated with differences in nicotine metabolism across African American, Native Hawaiian, Caucasian, Latino, and Japanese American smokers.^65^, ^66^ Analyses of the *UGT2A1/2* variants across populations may reveal differential risk to ovarian disease. In addition, expression and functional analysis in ovarian tissue needs to be accomplished to elucidate the impact on tissue homeostasis. In spite of the limitations of this study, these results provide new insight into proliferative and hormone target pathways that may represent important opportunities for the development of chemotherapeutic targets and intervention strategies.

## CONFLICT OF INTEREST

The authors have no conflicts of interest to disclose.
