# Supplementary material for: Evaluation of vitamin D biosynthesis and pathway target genes reveals UGT2A1/2 and EGFR polymorphisms associated with epithelial ovarian cancer in African American Women
Source: Cancer Med. 2019 Apr 18;8(5):2503–13. doi: 10.1002/cam4.1996 (PMC6536963; doi:10.1002/cam4.1996)
Supplement: Supplementary file 1 [file CAM4-8-2503-s001.doc]

| **Supplemental Table 1. AACES and OCAC Sites Included in the African Ancestry OncoArray Analysis** | | | | | | |
| --- | --- | --- | --- | --- | --- | --- |
|  | **EOC** | | | **HGSOC** | | |
|  | **Cases** | **Controls** | **Total** | **Cases** | **Controls** | **Total** |
| **Study Sites** | **n (%)** | **n (%)** | **n (%)** | **n (%)** | **n (%)** | **n (%)** |
| AAS – African American Cancer Epidemiology Study | 467 (61.9) | 563 (45.6) | 1030 (51.8) | 349 (65.0) | 563 (45.6) | 912 (51.5) |
| BEL – Belgium Ovarian Cancer Study | 0 (0.0) | 2 (0.2) | 2 (0.1) | 0 (0.0) | 2 (0.2) | 2 (0.1) |
| BVU – The BioVU DNA Repository | 8 (1.1) | 98 (7.9) | 106 (5.3) | 4 (0.7) | 98 (7.9) | 102 (5.8) |
| CAM – Cancer Research UK, Cambridge Research Institute | 1 (0.1) | 0 (0.0) | 1 (0.1) | 0 (0.0) | 0 (0.0) | 0 (0.0) |
| DKE – Duke University Clinic | 7 (0.9) | 0 (0.0) | 7 (0.4) | 3 (0.6) | 0 (0.0) | 3 (0.2) |
| DOV – Diseases of the Ovary and their Evaluation | 7 (0.9) | 27 (2.2) | 34 (1.7) | 4 (0.7) | 27 (2.2) | 31 (1.8) |
| HAW – Hawaii Ovarian Cancer Study | 0 (0.0) | 3 (0.2) | 3 (0.2) | 0 (0.0) | 3 (0.2) | 3 (0.2) |
| HOP – Hormones and Ovarian Cancer Prediction | 16 (2.1) | 21 (1.7) | 37 (1.9) | 10 (1.9) | 21 (1.7) | 31 (1.8) |
| LAX – Women's Cancer Program at the Samuel Oschin Comprehensive Cancer Institute | 18 (2.4) | 0 (0.0) | 18 (0.9) | 15 (2.8) | 0 (0.0) | 15 (0.9) |
| MAY – Mayo Clinic Ovarian Cancer Case-Control Study | 3 (0.4) | 5 (0.4) | 8 (0.4) | 2 (0.4) | 5 (0.4) | 7 (0.4) |
| MEC – Multiethnic Cohort Study | 10 (1.3) | 13 (1.1) | 23 (1.2) | 6 (1.1) | 13 (1.1) | 19 (1.1) |
| MOF – Moffitt Cancer Center Ovarian Cancer Study | 16 (2.1) | 14 (1.1) | 30 (1.5) | 13 (2.4) | 14 (1.1) | 27 (1.5) |
| MSK – Memorial Sloan Kettering Cancer Center | 8 (1.1) | 20 (1.6) | 28 (1.4) | 8 (1.5) | 20 (1.6) | 28 (1.6) |
| NCO – North Carolina Ovarian Cancer Study | 108 (14.3) | 160 (13.0) | 268 (13.5) | 76 (14.2) | 160 (13.0) | 236 (13.3) |
| NEC – New England Case-Control Study of Ovarian Cancer | 6 (0.8) | 5 (0.4) | 11 (0.6) | 6 (1.1) | 5 (0.4) | 11 (0.6) |
| NHS – Nurses’ Health Study I and II | 3 (0.4) | 0 (0.0) | 3 (0.2) | 3 (0.6) | 0 (0.0) | 3 (0.2) |
| NOR – University of Bergen, Haukeland University Hospital, Norway | 1 (0.1) | 0 (0.0) | 1 (0.1) | 0 (0.0) | 0 (0.0) | 0 (0.0) |
| NTH – Nijmegen Ovarian Cancer Study | 1 (0.1) | 0 (0.0) | 1 (0.1) | 0 (0.0) | 0 (0.0) | 0 (0.0) |
| ORE – Oregon Ovarian Cancer Registry | 1 (0.1) | 0 (0.0) | 1 (0.1) | 1 (0.2) | 0 (0.0) | 1 (0.1) |
| OVA – Ovarian Cancer in Alberta and British Columbia | 1 (0.1) | 0 (0.0) | 1 (0.1) | 0 (0.0) | 0 (0.0) | 0 (0.0) |
| PLC –Prostate-Lung-Colorectal and Ovarian Cancer Screening Trial | 5 (0.7) | 85 (6.9) | 90 (4.6) | 2 (0.4) | 85 (6.9) | 87 (4.9) |
| RMH – Royal Marsden Hospital Ovarian Cancer Study | 1 (0.1) | 0 (0.0) | 1 (0.1) | 1 (0.2) | 0 (0.0) | 1 (0.1) |
| RPC – Roswell Park Cancer Institute Ovarian Cancer Cohort | 4 (0.5) | 0 (0.0) | 4 (0.2) | 3 (0.6) | 0 (0.0) | 3 (0.2) |
| SEA – UK Studies of Epidemiology and Risk Factors in Cancer Heredity (SEARCH) Ovarian Cancer Study | 2 (0.3) | 1 (0.1) | 3 (0.2) | 1 (0.2) | 1 (0.1) | 2 (0.1) |
| SIS – The Sister Study | 8 (1.1) | 131 (10.6) | 139 (7.0) | 3 (0.6) | 131 (10.6) | 134 (7.6) |
| SOC – Southampton Ovarian Cancer Study | 1 (0.1) | 0 (0.0) | 1 (0.1) | 0 (0.0) | 0 (0.0) | 0 (0.0) |
| STA – Genetic Epidemiology of Ovarian Cancer | 12 (1.6) | 45 (3.6) | 57 (2.9) | 4 (0.7) | 45 (3.6) | 49 (2.8) |
| UCI – UC Irvine Ovarian Cancer Study | 0 (0.0) | 2 (0.2) | 2 (0.1) | 0 (0.0) | 2 (0.2) | 2 (0.1) |
| UHN – Princess Margaret Cancer Centre | 4 (0.5) | 0 (0.0) | 4 (0.2) | 2 (0.4) | 0 (0.0) | 2 (0.1) |
| UKO – UK Ovarian Cancer Population Study | 4 (0.5) | 10 (0.8) | 14 (0.7) | 2 (0.4) | 10 (0.8) | 12 (0.7) |
| USC – Los Angeles County Case-Control Studies of Ovarian Cancer | 31 (4.1) | 30 (2.4) | 61 (3.1) | 18 (3.4) | 30 (2.4) | 48 (2.7) |
| WMH – Westmead Institute for Cancer Research – Westmead Hospital | 1 (0.1) | 0 (0.0) | 1 (0.1) | 1 (0.1) | 0 (0.0) | 1 (0.1) |
| ***TOTAL*** | ***755*** | ***1235*** | ***1990*** | ***537*** | ***1235*** | ***1772*** |
