# Supplementary material for: Evaluation of vitamin D biosynthesis and pathway target genes reveals UGT2A1/2 and EGFR polymorphisms associated with epithelial ovarian cancer in African American Women
Source: Cancer Med. 2019 Apr 18;8(5):2503–13. doi: 10.1002/cam4.1996 (PMC6536963; doi:10.1002/cam4.1996)
Supplement: Supplementary file 2 [file CAM4-8-2503-s002.docx]

Supplemental Table 2: Power Calculations for EOC

|  |  | **African American (Cases=755, Controls=1,235)** | | | | | **Caucasian** | |
| --- | --- | --- | --- | --- | --- | --- | --- | --- |
| **Minor allele frequency (MAF)** | **Odds ratio** | **EOC**  **(5 SNPs)** | **EOC**  **(100 SNPs)** | **EOC**  **(500 SNPs)** | **EOC**  **(1000 SNPs)** | **EOC**  **(5000 SNPs)** | **EOC**  **(Cases=25,509,**  **Controls=40,941)** | **Serous**  **(Cases=16,013,**  **Controls=40,941)** |
| 0.05 | 1.05 | 0.014 | 0.001 | 0.0002 | 0.0001 | 0.0000 | 0.479 | 0.372 |
|  | 1.10 | 0.028 | 0.002 | 0.001 | 0.0003 | 0.0001 | 0.999 | 0.898 |
|  | 1.20 | 0.096 | 0.014 | 0.004 | 0.003 | 0.001 | 0.999 | 0.999 |
| 0.15 | 1.05 | 0.022 | 0.002 | 0.0004 | 0.0002 | 0.0001 | 0.876 | 0.761 |
|  | 1.10 | 0.065 | 0.008 | 0.002 | 0.001 | 0.0004 | 0.999 | 0.999 |
|  | 1.20 | 0.303 | 0.078 | 0.034 | 0.023 | 0.009 | 0.999 | 0.999 |
| 0.25 | 1.05 | 0.028 | 0.002 | 0.0006 | 0.0003 | 0.0001 | 0.965 | 0.898 |
|  | 1.10 | 0.097 | 0.014 | 0.005 | 0.003 | 0.001 | 0.999 | 0.999 |
|  | 1.20 | 0.459 | 0.157 | 0.078 | 0.057 | 0.026 | 0.999 | 0.999 |

Note: assuming prevalence rate of 0.001. For Caucasian look-ups, power calculations were done based on a nominal cutoff P=0.05.

Supplemental Table 3: Power Calculations for HGSOC

|  |  | **African American (Cases=537, Controls=1,235)** | | | | | **Caucasian** |
| --- | --- | --- | --- | --- | --- | --- | --- |
| **Minor allele frequency (MAF)** | **Odds ratio** | **HGSOC**  **(5 SNPs)** | **HGSOC**  **(100 SNPs)** | **HGSOC**  **(500 SNPs)** | **HGSOC**  **(1000 SNPs)** | **HGSOC**  **(5000 SNPs)** | **HGSOC**  **(Cases=13,037,**  **Controls=40,941)** |
| 0.05 | 1.05 | 0.014 | 0.001 | 0.0002 | 0.0001 | 0.0000 | 0.328 |
|  | 1.10 | 0.024 | 0.002 | 0.001 | 0.0003 | 0.0001 | 0.850 |
|  | 1.20 | 0.075 | 0.010 | 0.003 | 0.002 | 0.001 | 0.999 |
| 0.15 | 1.05 | 0.019 | 0.001 | 0.0003 | 0.0002 | 0.0000 | 0.697 |
|  | 1.10 | 0.052 | 0.006 | 0.002 | 0.001 | 0.0003 | 0.998 |
|  | 1.20 | 0.232 | 0.051 | 0.020 | 0.014 | 0.005 | 0.999 |
| 0.25 | 1.05 | 0.024 | 0.002 | 0.0005 | 0.0003 | 0.0001 | 0.849 |
|  | 1.10 | 0.076 | 0.010 | 0.003 | 0.002 | 0.001 | 0.999 |
|  | 1.20 | 0.358 | 0.102 | 0.047 | 0.033 | 0.014 | 0.999 |

Note: assuming prevalence rate of 0.001. For Caucasian look-ups, power calculations were done based on a nominal cutoff P=0.05.

Supplemental Table 4: Genes and gene regions related to Vitamin D metabolism

| **Gene** | **Genome Location (build 37)** | **Number of SNPs passing filters for analysis of EOC/HGSOC** |
| --- | --- | --- |
| *VDR* | chr12:48,235,320 – 48,298,814 | 288/234 |
| *EGFR* | chr7:55,086,678 – 55,279,262 | 963/824 |
| *UGT1A* (includes 1, 3, 4, 5, 6, 7, 8, 9, 10) | chr2:234,494,085 – 234,681,945 | 919/833 |
| *UGT2B* (includes 15, 17, 10, 11, 28, 4, 7) | chr4:69,402,902 - 70,391,732 | 6302/5674 |
| *UGT2A1/2* | chr4:70,454,135 – 70,518,967 | 433/413 |
| *CYP3A4/5* | chr7:99,245,813-99,381,262 | 411/375 |
| *CYP2R1* | chr11:14,899,551 – 14,913,874 | 17/15 |
| *CYP27B1* | chr12:58,156,117 – 58,160,976 | 4/4 |
| *CYP24A1* | chr20:52,769,985 – 52,790,516 | 113/106 |
| *CYP11A1* | chr15:74,630,103 – 74,660,081 | 90/82 |
| *GC* | chr4:72,607,410 – 72,671,237 | 296/282 |
