# Supplementary material for: Evaluation of vitamin D biosynthesis and pathway target genes reveals UGT2A1/2 and EGFR polymorphisms associated with epithelial ovarian cancer in African American Women
Source: Cancer Med. 2019 Apr 18;8(5):2503–13. doi: 10.1002/cam4.1996 (PMC6536963; doi:10.1002/cam4.1996)
Supplement: Supplementary file 3 [file CAM4-8-2503-s003.docx]

| **Supplemental Table 5: Notable gene region SNP associations in African American OncoArray EOC analysis^a^, 755 cases and 1235 controls (p<0.01)** | | | | |
| --- | --- | --- | --- | --- |
| ***Nearest gene(s)***  **SNP ID (Effect / other allele)** | **Effect Allele Frequency** | **OR** | **(95% CI)** | **p-value** |
| ***VDR*** |  |  |  |  |
| rs79428807 (G/A) | 0.05 | 0.6 | (0.43, 0.87) | 0.006 |
| rs186107244 (T/C) | 0.02 | 2.0 | (1.20, 3.30) | 0.0097 |
| ***UGT2B*4** |  |  |  |  |
| rs72025959 (GAA/GA) | 0.16 | 0.7 | (0.58, 0.83) | 6.3X10-5 |
| rs10011506 (G/A) | 0.84 | 1.4 | (1.20, 1.70) | 7.1X10-5 |
| rs1560606 (A/C) | 0.84 | 1.4 | (1.20, 1.70) | 7.5 X10-5 |
| rs13136057 (A/T) | 0.84 | 1.4 | (1.20, 1.70) | 7.6X10-5 |
| rs1817904 (T/C) | 0.84 | 1.4 | (1.19, 1.70) | 7.7X10-5 |
| rs6600776 (T/G) | 0.84 | 1.4 | (1.19, 1.70) | 7.9X10-5 |
| rs2082335 (C/T) | 0.84 | 1.4 | (1.19, 1.70) | 8.1X10-5 |
| rs71205987 (A/AT) | 0.84 | 1.4 | (1.19, 1.70) | 8.1X10-5 |
| rs1594587 (A/T) | 0.84 | 1.4 | (1.19, 1.69) | 8.3X10-5 |
| rs2642873 (T/C) | 0.84 | 1.4 | (1.19, 1.69) | 8.8X10-5 |
| rs2195842 (T/A) | 0.83 | 1.4 | (1.18, 1.66) | 0.0001 |
| rs2009889 (C/T) | 0.83 | 1.4 | (1.18, 1.66) | 0.0001 |
| rs2010048 (G/A) | 0.83 | 1.4 | (1.18, 1.65) | 0.0001 |
| rs2331748 (T/G) | 0.82 | 1.4 | (1.18, 1.65) | 0.0001 |
| rs1594586 (T/C) | 0.83 | 1.4 | (1.18, 1.65) | 0.0001 |
| rs2082338 (G/A) | 0.83 | 1.4 | (1.18, 1.65) | 0.0001 |
| rs33997604 (TTTG/T) | 0.82 | 1.4 | (1.18, 1.65) | 0.0001 |
| rs2736442 (G/A)^a^ | 0.84 | 1.4 | (1.18, 1.68) | 0.0001 |
| rs1594585 (G/A) | 0.83 | 1.4 | (1.18, 1.65) | 0.0001 |
| rs113361105 (GT/G) | 0.82 | 1.4 | 1.17, 1.65) | 0.0001 |
| rs2331747 (A/G) | 0.82 | 1.4 | (1.17, 1.65) | 0.0001 |
| rs2195843 (A/G) | 0.09 | 0.7 | (0.55, 0.86) | 0.0007 |
| ***UGT2B10*** |  |  |  |  |
| rs12503658 (A/G) | 0.49 | 0.8 | (0.70, 0.91) | 0.0009 |
| ***UGT1A*** |  |  |  |  |
| rs1604144(T/C)^a^ | 0.43 | 1.2 | (1.07, 1.39) | 0.003 |
| rs28900068 (A/G) | 0.04 | 1.6 | (1.15, 2.23) | 0.005 |
| rs33979061 (C/A) | 0.08 | 1.5 | (1.12, 1.91) | 0.005 |
| rs28898579 (C/G) | 0.03 | 1.6 | (1.14, 2.25) | 0.007 |
| rs3832043 (A/AT) | 0.56 | 1.2 | (1.05, 1.36) | 0.007 |
| rs113090807 (G/GGTTT) | 0.04 | 1.6 | (1.12, 2.20) | 0.008 |
| rs45566031 (G/A) | 0.04 | 1.6 | (1.12, 2.21) | 0.008 |
| rs17854828 (T/C) | 0.16 | 1.3 | (1.06, 1.50) | 0.008 |
| rs28898575 (T/C) | 0.04 | 1.6 | (1.12, 2.20) | 0.009 |
| rs147695639 (A/AT) | 0.16 | 1.3 | (1.06, 1.50) | 0.009 |
| rs28970018 (A/G) | 0.04 | 1.6 | (1.12, 2.21) | 0.009 |
| ***EGFR*** |  |  |  |  |
| rs114972508 (C/T) | 0.04 | 1.7 | (1.23, 2.47) | 0.002 |
| rs41407249 (G/A) | 0.054 | 1.6 | (1.17, 2.12) | 0.003 |
| rs147112830 (A/G) | 0.023 | 1.9 | (1.21, 3.01) | 0.005 |
| rs79842417 (A/G) | 0.056 | 1.5 | (1.12, 2.01) | 0.007 |
| rs115967098 (A/C) | 0.043 | 1.5 | (1.12, 2.16) | 0.009 |
| ***CYP3A*** |  |  |  |  |
| rs45560331 (G/A) | 0.05 | 1.5 | (1.11, 2.04) | 0.008 |
| ^a^Genotyped | | | | |

| **Supplemental Table 7. African American OncoArray EOC and HGSOC analysis results for SNPs with previous report of EOC association in the VDR gene, 755 cases and 1235 controls** | | | | | | | | |
| --- | --- | --- | --- | --- | --- | --- | --- | --- |
|  | | **EOC** | | | | **HGSOC** | | |
| **VDR SNP ID (Effect / other allele)** | **Effect Allele Frequency** | **OR** | **95% CI** | | **p-value** | **OR** | **95% CI** | **p-value** |
| rs7975232 (Apa1) (A/C) | 0.63 | 1.01 | (0.88, 1.16) | | 0.90 | 0.99 | (0.85, 1.16) | 0.99 |
| rs2228570 (FokI) (G/A)^a^ | 0.80 | 0.99 | (0.85, 1.17) | | 0.99 | 0.98 | (0.82, 1.17) | 0.82 |
| rs7305032 (A/G)^b^ | 0.70 | 0.99 | (0.86, 1.14) | | 0.89 | 1.06 | (0.90, 1.25) | 0.50 |
| rs154410 (BsmI) (T/C)^a^ | 0.31 | 1.04 | (0.90, 1.2) | | 0.56 | 1.10 | (0.94, 1.28) | 0.24 |
| rs731236 (TaqI) (G/A) | 0.30 | 0.98 | (0.84, 1.14) | | 0.80 | 1.03 | (0.87, 1.22) | 0.71 |
| rs2239179 (C/T)^a,b^ | 0.33 | 1.04 | (0.90, 1.19) | | 0.60 | 0.72 | (0.53, 0.98) | 0.04 |
| rs3782905 (C/G)^a,b^ | 0.24 | 0.96 | (0.82, 1.12) | | 0.60 | 0.99 | (0.84, 1.18) | 0.96 |
| rs2248098 (G/A)^a,b^ | 0.50 | 1.10 | (0.97, 1.25) | | 0.13 | 1.09 | (0.23, 0.94) | 0.24 |
| rs2525044 (G/A)^b^ | 0.78 | 1.08 | (0.92, 1.27) | | 0.34 | 1.07 | (0.89, 1.27) | 0.47 |
| rs7971418 (A/C)^b^ | 0.56 | 0.99 | (0.87, 1.14) | | 0.99 | 1.01 | (0.87, 1.18) | 0.87 |
| rs987849 (A/G)^b^ | 075 | 1.13 | (0.96, 1.31) | | 0.13 | 1.13 | (0.95, 1.34) | 0.17 |
| ^a^Genotyped  b[11] |  | |  |  | |  |  |  |
