# Supplementary material for: Evaluation of vitamin D biosynthesis and pathway target genes reveals UGT2A1/2 and EGFR polymorphisms associated with epithelial ovarian cancer in African American Women
Source: Cancer Med. 2019 Apr 18;8(5):2503–13. doi: 10.1002/cam4.1996 (PMC6536963; doi:10.1002/cam4.1996)
Supplement: Supplementary file 4 [file CAM4-8-2503-s004.docx]

| **Supplemental Table 6. Notable gene region SNP associations in African American OncoArray HGSOC analysis (p<0.01), 537 cases and 1235 controls** | | | | |
| --- | --- | --- | --- | --- |
| ***Nearest gene(s)***  **SNP ID (Effect / other allele)** | **Effect Allele Frequency** | **OR** | **95% CI** | **p-value** |
| ***UGT1A*** | | | | |
| rs33979061 (C/A) | 0.08 | 1.6 | (1.20, 2.19) | 0.001 |
| rs17863773 (C/T)^a^ | 0.14 | 1.4 | (1.11, 1.68) | 0.003 |
| rs6706988 (G/A) | 0.08 | 1.5 | (1.14, 1.90) | 0.003 |
| rs34352422 (C/CT) | 0.46 | 1.2 | (1.07, 1.45) | 0.005 |
| rs147695639 (A/AT) | 0.16 | 1.3 | (1.09, 1.61) | 0.005 |
| rs17854828 (T/C) | 0.16 | 1.3 | (1.09, 1.61) | 0.005 |
| rs12053462 (G/A) | 0.13 | 1.4 | (1.09, 1.69) | 0.005 |
| rs78535700 (T/TATCAGGTC) | 0.12 | 1.4 | (1.09, 1.70) | 0.005 |
| rs4663325 (T/C) | 0.23 | 1.3 | (1.07, 1.50) | 0.006 |
| rs36043462 (A/AT) | 0.23 | 1.3 | (1.07, 1.50) | 0.006 |
| rs4663877 (A/G) | 0.12 | 1.3 | (1.09, 1.68) | 0.006 |
| rs4663871 (A/G) | 0.14 | 1.3 | (1.08, 1.62) | 0.006 |
| rs17862856 (A/G) | 0.14 | 1.3 | (1.08, 1.62) | 0.006 |
| rs10207520 (G/A) | 0.12 | 1.3 | (1.09, 1.68) | 0.007 |
| rs17864684 (A/G) | 0.14 | 1.3 | (1.08, 1.64) | 0.007 |
| rs4485562 (A/G) | 0.13 | 1.3 | (1.08, 1.67) | 0.007 |
| rs12474980 (A/G) | 0.13 | 1.3 | (1.08, 1.67) | 0.007 |
| rs12476197 (C/G) | 0.13 | 1.3 | (1.08, 1.67) | 0.007 |
| rs17868325 (G/T) | 0.13 | 1.3 | (1.08, 1.67) | 0.007 |
| rs6753317 (A/T) | 0.13 | 1.3 | (1.08, 1.67) | 0.007 |
| rs11330313 (A/AG) | 0.14 | 1.3 | (1.08, 1.61) | 0007 |
| rs17864686 (A/G) | 0.13 | 1.3 | (1.08, 1.67) | 0.007 |
| rs12474215 (T/A) | 0.12 | 1.3 | (1.08, 1.67) | 0.007 |
| rs12472689 (T/C)^a^ | 0.12 | 1.3 | (1.08, 1.67) | 0.007 |
| rs143064337 (T/G) | 0.07 | 1.5 | (1.11, 1.93) | 0.007 |
| rs58008965 (C/CTT) | 0.31 | 1.2 | (1.05, 1.43) | 0.008 |
| rs1604144 (T/C) | 0.43 | 1.2 | (1.05, 1.41) | 0.009 |
| rs115061010 (T/C) | 0.07 | 1.4 | (1.10, 1.93) | 0.009 |
| ***UGT2B15*** |  |  |  |  |
| rs143827831 (G/A) | 0.07 | 1.8 | (1.29, 2.45) | 0.0004 |
| rs62298438 (C/T) | 0.07 | 1.8 | (1.29, 2.46) | 0.0004 |
| ***UGT2B4*** |  |  |  |  |
| rs72025959 (GAA/GA) | 0.17 | 0.7 | (0.50, 0.86) | 0.0006 |
| rs10011506 (G/A) | 0.84 | 1.4 | (1.16, 1.71) | 0.0006 |
| rs1560606 (A/C) | 0.84 | 1.4 | (1.15, 1.70) | 0.0007 |
| rs13136057 (A/T) | 0.84 | 1.4 | (1.15, 1.70) | 0.0007 |
| rs1817904 (T/C) | 0.84 | 1.4 | (1.15, 1.70) | 0.0007 |
| rs6600776 (T/G) | 0.84 | 1.4 | (1.15, 1.70) | 0.0007 |
| rs2082335 (C/T) | 0.84 | 1.4 | (1.15, 1.70) | 0.0007 |
| rs71205987 (A/AT) | 0.84 | 1.4 | (1.15, 1.70) | 0.0007 |
| rs1594587 (A/T) | 0.84 | 1.4 | (1.15, 1.70) | 0.0007 |
| rs2642873 (T/C) | 0.84 | 1.4 | (1.15, 1.70) | 0.0007 |
| rs2736442 (G/A)^a^ | 0.84 | 1.4 | (1.15, 1.70) | 0.0009 |
| ***EGFR*** |  |  |  |  |
| rs75546857 (G/A) | 0.04 | 1.7 | (1.27, 2.31) | 0.0004 |
| rs2286962 (C/A) | 0.06 | 1.7 | (1.27, 2.31) | 0.0004 |
| rs115391410 (C/T) | 0.06 | 1.7 | (1.27, 2.31) | 0.0004 |
| rs116347781 (T/C) | 0.03 | 1.9 | (1.26, 2.86) | 0.002 |
| rs79842417 (A/G) | 0.05 | 1.6 | (1.15, 2.23) | 0.005 |
| rs17289232 (C/A) | 0.10 | 1.4 | (1.10, 1.80) | 0.006 |
| rs113410025 (T/C) | 0.01 | 1.4 | (1.10, 1.80) | 0.006 |
| rs17290755 (T/C) | 0.02 | 1.9 | (1.18, 3.07) | 0.008 |
| rs17337563 (A/G) | 0.02 | 1.9 | (1.18, 3.06) | 0.008 |
| rs17290792 (G/C) | 0.02 | 1.9 | (1.18, 3.07) | 0.008 |
| ^a^Genotyped |  |  |  |  |
