# Supplementary material for: Evaluation of vitamin D biosynthesis and pathway target genes reveals UGT2A1/2 and EGFR polymorphisms associated with epithelial ovarian cancer in African American Women
Source: Cancer Med. 2019 Apr 18;8(5):2503–13. doi: 10.1002/cam4.1996 (PMC6536963; doi:10.1002/cam4.1996)
Supplement: Supplementary file 5 [file CAM4-8-2503-s005.docx]

| **Supplemental Table 8. Associations of significant study SNPs in Caucasians from OCAC database** | | | | |
| --- | --- | --- | --- | --- |
| **Gene** | **SNP** | **Chromosome position**  **(build 37)** | **Serous**  **OR (SE) Whites** | **P-value** |
| UGT2A1/2 | rs10017134 | 4:70456771-7045727 | OR: 0.986 (0.95-1.02) | 0.4414 |
| UGT2A1/2 | rs2288741 | 4:69589760 | OR: 0.983 (0.95-1.02) | 0.3508 |
| EGFR | rs114972508 | 7:55088259-55088758 | NA | NA |
| **Gene** | **SNP** | **Chromosome position**  **(build 37)** | **High grade Serous**  **OR (SE) Whites** | **P-value** |
| UGT2A1/2 | rs10017134 | 4:70456771-7045727 | OR: 0.986 (0.95-1.02) | 0.4387 |
| UGT2A1/2 | rs2288741 | 4:69589760 | OR: 0.944 (0.96-1.03) | 0.3505 |
| EGFR | rs114972508 | 7:55088259-55088758 | NA | NA |
